# The extent and nature of television food advertising to children in Xi’an, China

**DOI:** 10.1186/s12889-016-3468-0

**Published:** 2016-08-11

**Authors:** Danyang Li, Ting Wang, Yue Cheng, Min Zhang, Xue Yang, Zhonghai Zhu, Danli Liu, Wenfang Yang, Lingxia Zeng

**Affiliations:** 1 Department of Epidemiology & Biostatistics, School of Public Health, Xi’an Jiaotong University Health Science Center, Xi’an, Shaanxi Province, 710061 China; 2 Cardio-cerebrovascular Diseases Hospital of Ningxia Medical University, Yinchuan, Ningxia Province, 750002 China; 3 The First Affiliated Hospital of Xi’an Jiaotong University, Xi’an, Shaanxi Province, 710061 China

**Keywords:** Children obesity, Food advertising, Food promotion, Core food

## Abstract

**Background:**

To explore the extent and nature of television food advertising especially unhealthy food advertising to primary school children in Xi’an, China.

**Methods:**

Television data were recorded for 2 weekdays and 2 weekend days between 6:00 and 22:00 during May and June in 2012 from a total of five television channels most popular with children in Xi’an. Pearson *χ*^2^ tests and logistic regression were applied to determine differences in the proportion of healthy food, unhealthy food and miscellaneous food advertisements for different channels, programs, dates, viewing periods and the use of persuasive marketing tactics.

**Results:**

Of the 5527 advertisements transcribed, 25.5 % were for food, among which 48.1 % were considered to be unhealthy. The frequency of food advertisements was 6 per hour per channel, including 3 unhealthy food advertisements. Compared with healthy and miscellaneous food advertisements, more unhealthy food advertisements were shown during afternoon, weekends and children’s non-peak viewing times as well as on children’s television channels, central television channels and non-children’s programmes. Unhealthy foods contributed the highest proportion of all food advertisements containing promotional characters (51.7 %) and premium offers (59.1 %). Both promotional characters and premium offers appeared more on non-children’s television channels.

**Conclusions:**

The majority of food advertisements were for unhealthy food. More unhealthy food ads were shown in children’s non-peak time and afternoon as well as non-children’s channels. More children-oriented persuasive marketing tactics were used in unhealthy food ads especially in non-children’s channels. Therefore, intervening in the entrance of unhealthy foods into the market and establishing regulations related to food advertising especially unhealthy food advertisements are important strategies to prevent children’s exposure to unhealthy food and childhood obesity.

**Electronic supplementary material:**

The online version of this article (doi:10.1186/s12889-016-3468-0) contains supplementary material, which is available to authorized users.

## Background

Obesity is a multi-factor disease resulting in the accumulation and increase of excess body fat [1]. Globally, almost 1.5 billion people are overweight, including more than 40 million children under the age of five [2]. Within the last 20–30 years, the number of overweight and obese children has increased rapidly throughout the world [3]. In China during 1985–2000, the prevalence of overweight in children aged 7–18 years increased 28 fold, and obesity increased 4 fold [4]. In Beijing and Shanghai, the prevalence of overweight and obesity in 7–12 year olds approached 29 % for boys and 15–17 % for girls in 2000 [5]. In addition, it was reported that 16.3 % of adolescents including 19.4 % of boys and 13.2 % of the girls were overweight or obese in Xi’an [6]. Hence, the increasing level of obesity prevalence in children, combined with the potential effects that reach into adulthood [7, 8] has become a public health problem at present and probably in the near future.

Food marketing has become an influence on children’s dietary patterns [9]. Not merely supermarkets but also television (TV) viewing, convenience stores, and the internet compose the bulk of sites for children to be exposed to food marketing. Recently, the development and expansion of unhealthy food marketing has become an important environmental factor leading to childhood obesity, which automatically triggers the purchase and consumption behaviours of children such as nutrition knowledge, preferences, purchase behaviour, and diet-related health surrounding food [10, 11]. Moreover, the increase of inactive indoor lifestyles including watching TV, playing video games, and using the internet, supports the possibility of more energy-dense nutrient-poor dietary intakes underlying the dramatic rise in childhood obesity [9, 12, 13].

TV advertising, as a popular public media plays an important role in children food marketing. In America, the Federal Trade Commission reported that 2.1 billion dollars were spent by 44 food and beverage companies on promoting their products to children and adolescents [14], in which TV food advertising aimed at children and teenagers costs almost 1 billion dollars [14]. Meanwhile, the cost of fast-food restaurant TV advertising aimed at children increased 59.5 % during 2006–2009 [15]. All these data indicate that food advertising is gradually expanding and is gaining a more important role in influencing children’s choice of food. Watching TV for a long period of time will increase the tendency toward and possibility of childhood obesity due to some unhealthy dietary behaviours, including more consumption of high-fat foods, fast foods and sugar-sweetened beverages [12, 13, 16]. In China, children and adolescents who reported paying attention to commercials were more likely to request snacks and buy snacks seen on TV, so attention to TV commercials for snack foods may be one of the factors affecting the increase in obesity among children and adolescents [17]. At the same time, the propaganda of energy-dense nutrient-poor foods together with celebrity endorsement and brand effects in TV advertising has influenced children on cognitive choice and attention to food [18]. A previous study indicated that children in developing countries who were younger than twelve did not have enough cognitive system and nutrition knowledge to judge and identify the intention of advertising food promotion, so they tended to choose advertised foods and increase the risk of obesity indirectly [19].

A recent study [20] in which our team was partly involved has provided a brief summary on the rate of all ads and food ads as well as food ads containing persuasive marketing techniques across six sites in the Asia-Pacific region. However, differences among regions, including advertisement types, distribution and promotion techniques still exist. In addition, the distribution difference and the broadcast pattern among different food type ads and ersuasive marketing techniques by different viewing categories have not been understood in Xi’an, China. Therefore, based on the study by Kelly et al. [20] and combined local conditions of Xi’an, which is the largest city in the northwest China, this study aims to more exhaustively explore and detail the characteristics of food advertising on TV in children’s different viewing times, channels and programs, and assess the use of marketing tactics and the features of children’s exposure to unhealthy food advertising. We aim to explain a potential relation of different food ads in different programmes, channels, and times, lay the foundation for further research and provide specific recommendations for local government to formulate laws and regulations regarding food advertising on TV.

## Methods

A study by Kelly et al. [20] has reported the methods including the determination of the TV record, randomized selection of TV data and advertisement code standards. In addition to the above, a simple description is therefore provided, as follows.

### Sampling

Before TV data recording, a questionnaire survey aiming at 12 year-old children was conducted in an elementary school of Xi’an. According to the results from the preliminary research, a total of five TV channels that children like most were chosen respectively on weekdays [China Central Television-14 (CCTV-14), Hunan Television (Hunan TV) and Shaanxi Television (Shaanxi TV)] and weekend days [China Central Television-5 (CCTV-5), China Central Television-6 (CCTV-6), CCTV-14] for our further analysis. For all the five TV channels, TV data were acquired from two weekdays (Thursday and Friday) and two weekend days between 6:00 and 22:00 during May and June of 2012. All TV programmes and TV ads from each channel were recorded on hard disk, with all channels simultaneously recorded if possible.

### Coding

TV data were coded for channel name, date, day of the week, programme name during which the advertisement was shown, programme category, programme starting time, time slot of the programme (in half hour increments), and advertisement product type. Meanwhile, food advertisement data included company name, advertised products, food code, persuasive marketing skills and their description.

We developed a number of viewing categories with which to analyze product placement. The times during which the children watched TV were categorized as peak viewing time and non-peak viewing time, as well as morning, afternoon and evening. The children’s peak viewing time was defined as a period when most children were watching TV. In this study, it was defined as 17:00–20:00 based on the results of the preliminary research, while other periods were considered as non-peak viewing time. Moreover, it was specified that morning time was 6:00–12:30, afternoon time was 12:30–18:30, and evening time was 18:30–22:00. Channels were divided into central/local TV channels and children’s/non-children’s TV channels. Of the five channels, CCTV-5, −6, and −14 are central TV channels, and the others are local TV channels. CCTV-14 was the only children’s channel, the remaining were considered as non-children’s TV channels. Moreover, fifteen programme types were transcribed and classified as children’s programmes and non-children’s programmes (the other 14 programme types excluded children’s programmes) (Additional file 1: Table S1). Twenty-three different product types in TV advertising were also defined (Additional file 1: Table S2). The food categories in food ads on TV were classified as core and healthy foods (nutrient dense, low in energy), non-core and unhealthy foods (high in undesirable nutrients or energy) and miscellaneous foods (such as baby and toddler milk formula, dietary supplements and food additives) (Additional file 1: Table S3). In addition, it is important to note that unlike other countries, full-cream milks are still considered as core foods in China. Furthermore, the persuasive marketing tactics were divided into promotional characters including celebrities, spokes people/branded characters, licensed characters, sports figures and premium offers like giveaways, vouchers and rebates to promote foods [20]. All the results in this study are based on the above standards.

To certify data integrity and reliability, the recorders were trained before the formal investigation. Two of them completed the data conversion together using unified methods and checked errors for each other after conversion. During data cleaning, suspicious data were verified, and the original recordings were examined to ensure data accuracy.

### Analysis and reliability

All data were checked and cleaned prior to analysis. Diagrams were produced by Microsoft Excel 2010. Data were analysed with STATA version 12.0. The extent of food advertising were described as the rate of ads by channel, date and time per hour. Differences in the proportion of food ads for different viewing channels, dates, times and programme categories were applied using Pearson *χ*^2^ tests. Univariate and multivariate logistic regression analyses were used to assess the characteristics of food advertising and persuasive marketing techniques. Significant results were considered at α = 0.05.

Cohen’s k statistic was used for evaluating the correlation between groups’ coding. As a previous study reported [20], the k statistic for the classification of ads (k: 0.82) as for either food or non-food products was almost perfect.

## Results

### Overall food advertising

**Fig. 1:**
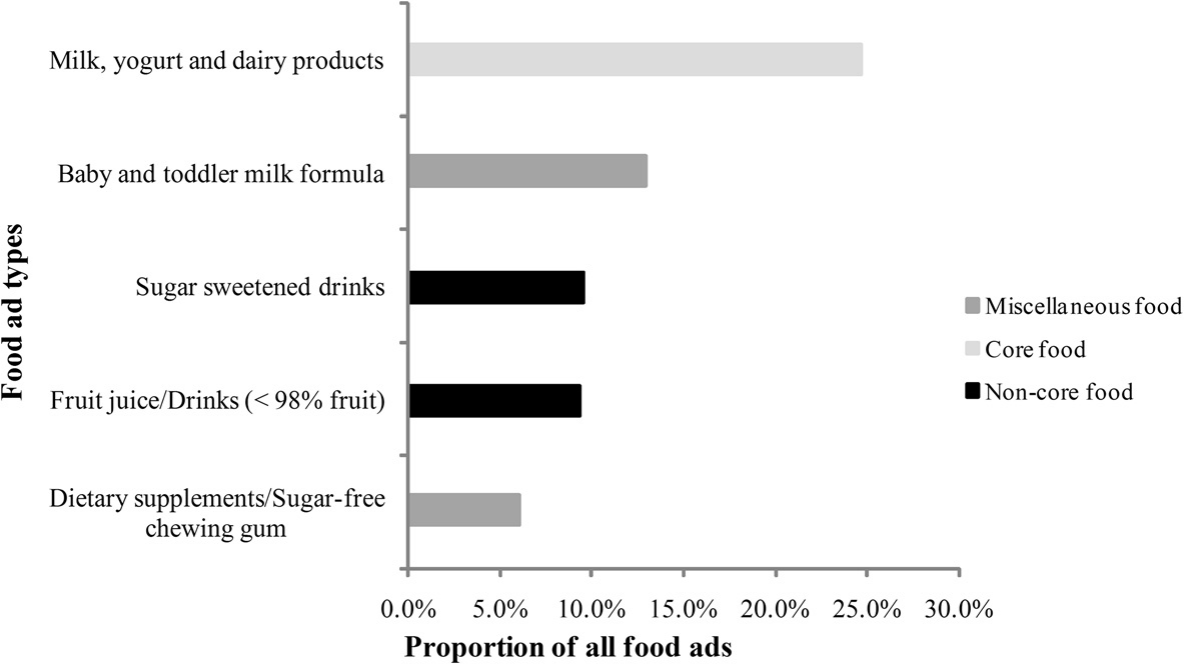
Top five advertised food products

In total, we obtained 192 h of TV data. Across all channels, 5527 ads were transcribed, of which the top five advertised products were retail food and drink, channel promotions, toiletries, clothes/shoes and public service (general), which accounted, respectively, for 25.5, 14.8, 14.0, 11.6 and 8.4 % of the ads. Most of the food ads were for non-core food contributing 48.1 % of all food ads. Milk, yoghurt and dairy products were the most frequently advertised foods. Sugar-sweetened drinks, fruit juice/drinks (<98 % fruit) were the most unhealthy foods in the ads, and the top five advertised food products of all food ads were shown in Fig. 1.

In the next sections we analyzed first the rate of food advertising per hour, second the distribution difference among three food type ads by different viewing categories, third how non-core food and total food ads varied by different viewing categories, and finally the use of what we term persuasive techniques in the advertising.

### The rate of food advertising per hour by channel, date and time

The overall rate of food advertising across the sample was 6 food ads per hour per channel including 3 unhealthy food ads, and the rate of food advertising on different channels per hour presented a significant difference based on our results and study by Kelly et al. [20], as shown in Table 1. Central TV channels, especially CCTV-14, broadcast the highest number of total ads, food ads, non-core food ads and food ads with promotional characters and premium offers per hour compared with other channels. Moreover, at different viewing dates and times, a disparity also existed in the number of food ads per hour per channel. The frequency of food ads was higher on weekends, particularly on Sunday, than on other days. Non-peak and evening were the two periods during which food ads appeared the most frequently, compared to peak time and morning or afternoon.

**Table 1:** The rate of food advertising by channel, date and time (per hour)

	Total ads	Food ads	Non-core food ads	Food ads with promotional characters	Food ads with premium offers
Channels					
Central TV channels	30.9	8.8	4.1	3.3	1.1
Local TV channels	15.1	3.0	1.6	1.3	0.2
Children’s channels	33.0	7.5	2.6	1.9	1.3
Non-children’s channels	26.6	7.3	4.0	3.3	0.5
Dates					
Weekdays	29.9	6.0	2.7	2.2	0.7
Weekends	27.7	8.7	4.4	3.5	0.9
Times					
Peak time	33.0	3.2	3.3	3.5	0.9
Non-peak time	27.8	7.1	3.3	3.7	0.8
Morning	25.8	6.0	2.9	2.5	0.7
Afternoon	28.1	7.3	3.9	2.4	0.7
Evening	35.5	9.9	4.2	4.1	1.2

*Ads* advertisements

### Food ads by viewing channel, date, time and programme category (Table 2)

**Table 2:** Food advertisements by viewing channel, date, time and programme category

	Core foods		Non-core foods		Miscellaneous foods		Total	
	*n*	%	*n*	%	*n*	%	*n*	%
Channels								
Central TV channels	316	30.0	490	46.5	247	23.5	1053	100.0
Local TV channels	108	30.3	190	53.4	58	16.3	356	100.0
Children’s channels	189	39.5	168	35.2	121	25.3	478	100.0
Non-children’s channels	235	25.2	512	55.0	184	19.8	931	100.0
Dates								
Weekdays	197	34.4	261	45.6	114	20.0	572	100.0
Weekends	227	27.1	419	50.1	191	22.8	837	100.0
Times								
Peak time	110	36.0	118	38.7	77	25.3	305	100.0
Non-peak time	314	28.4	562	50.9	228	20.7	1104	100.0
Morning	136	29.1	224	48.0	107	22.9	467	100.0
Afternoon	161	30.5	281	53.2	86	16.3	528	100.0
Evening	127	30.7	175	42.3	112	27.0	414	100.0
Programmes								
Children’s programmes	189	39.5	168	35.2	121	25.3	478	100.0
Non-children’s programmes	235	25.2	512	55.0	184	19.8	931	100.0
Total	424	30.1	680	48.3	305	21.7	1409	100.0

*Ads* advertisements

The total number of ads for different food types were influenced by channel, date, viewing time and programme. When comparing ads for the three types of food according to different factors, the differences were statistically significant (central/non-central TV channels: *χ*^2^ = 8.896, *P* = 0.012, children’s/non-children’s TV channels: *χ*^2^ = 51.733, *P* < 0.001; dates: *χ*^2^ = 8.743, *P* = 0.013; peak/non-peak time: *χ*^2^ = 14.336, *P* = 0.001; afternoon/morning, evening: *χ*^2^ = 18.939, *P* = 0.001; programmes: *χ*^2^ = 51.733, *P* < 0.001). Local TV channels accounted for the highest percentage of non-core food ads (53.4 %) and non-children’s channels broadcast the most non-core food ads (55.0 %). On weekends, more non-core food ads (50.1 %) were screened compared with weekdays (45.6 %). Meanwhile, during different time periods, more unhealthy food ads were screened during children’s non-peak viewing time (50.9 %) and afternoon (53.2 %) than during children’s peak viewing time (38.7 %) and morning (48.0 %) or evening (42.3 %). Non-children’s programmes accounted for the highest proportion of non-core food ads (55.0 %).

### The broadcast characteristics of total food and non-core food advertising in different viewing categories

According to Table 3, a significantly higher proportion of food ads were aired during weekends [31.5 %, OR = 1.28, 95 % CI (1.04, 1.57)] and afternoon or evening [afternoon: OR = 1.21, 95 % CI (1.04, 1.41); evening: OR = 1.46, 95 % CI (1.22, 1.73)] than during weekdays and morning. Among the different channels, local TV channels contained fewer food ads [19.6 %, OR = 0.53, 95 % CI (0.41, 0.69)], while more food ads were aired on non-children’s TV channels [55.0 %, OR = 1.75, 95 % CI (1.47, 2.08)]. Additionally, more non-core food ads appeared on weekends [OR = 1.18, 95 % CI (0.80, 1.72)] and a larger number of them fell during afternoon and non-peak time compared to morning [afternoon: OR = 1.41, 95 % CI (1.09, 1.83)] and peak time [OR = 1.60, 95 % CI (1.92, 2.16)]. Among the different channels, non-core food ads were distributed more on local TV [OR = 1.11, 95 % CI (0.70, 1.77)] and non-children’s TV channels [OR = 2.15, 95 % CI (1.59, 2.91)], though the difference was significant only for children’s/non-children’s TV channels.

**Table 3:** Characteristics of food advertising

	*N* (%)	The proportion of food ads among all ads			The proportion of non-core food ads among food ads		
		*n* (%)	OR (95 % CI)	Adjusted OR (95 % CI)	*n* (%)	OR (95 % CI)	Adjusted OR (95 % CI)
Dates							
Weekdays	2872 (52.0)	572 (19.9)	1.00 (−−)	1.00 (−−)	261 (45.6)	1.00 (−−)	1.00 (−−)
Weekends	2655 (48.0)	837 (31.5)	1.85 (1.64,2.09)*	1.28 (1.04,1.57)*	419 (50.1)	1.19 (0.97,1.48)	1.18 (0.80,1.72)
Times							
Peak time	1188 (21.5)	305 (25.7)	1.00 (−−)	1.00 (−−)	118 (38.7)	1.00 (−−)	1.00 (−−)
Non-peak time	4339 (78.5)	1104 (51.4)	0.99 (0.85,1.14)	1.11 (0.94,1.31)	562 (50.9)	1.64 (1.27,2.13)*	1.60 (1.19,2.16)*
Morning	2013 (36.4)	457 (23.2)	1.00 (−−)	1.00 (−−)	224 (48.0)	1.00 (−−)	1.00 (−−)
Afternoon	2021 (36.6)	528 (26.1)	1.17 (1.01, 1.35)*	1.21 (1.04, 1.41)*	281 (53.2)	1.23 (0.96, 1.58)	1.41 (1.09, 1.83)*
Evening	1493 (27.0)	414 (27.7)	1.27 (1.09,1.48)*	1.46 (1.22,1.73)*	175 (42.3)	0.79 (0.61,1.04)	1.06 (0.78,1.44)
Channels							
Central TV channels	3712 (67.2)	1053 (33.2)	1.00 (−−)	1.00 (−−)	490 (46.5)	1.00 (−−)	1.00 (−−)
Local TV channels	1815 (32.8)	356 (19.6)	0.62 (0.54,0.71)*	0.53 (0.41,0.69)*	190 (53.4)	1.32 (1.03,1.67)*	1.11 (0.70,1.77)
Children’s channels	2120 (38.4)	478 (22.6)	1.00 (−−)	1.00 (−−)	168 (35.2)	1.00 (−−)	1.00 (−−)
Non-children’s channels	3407 (61.6)	931 (27.3)	1.29 (1.14,1.47)*	1.75 (1.47,2.08)*	512 (55.0)	2.25 (1.80,2.83)*	2.15 (1.59,2.91)*

*Ads* advertisements

*: *P* < 0.05

### The broadcast characteristics of persuasive marketing techniques in different viewing categories

Overall, 38.5 and 10.9 % of all food ads contained promotional characters and premium offers. Non-core foods contributed the highest proportion of all food ads containing promotional characters (51.7 %) and premium offers (59.1 %). As shown in Table 4, the proportion of food ads containing promotional characters in non-children’s TV channels was higher than on children’s TV channels [OR = 2.64, 95 % CI (1.91, 3.66)]. Moreover, it was shown that fewer food ads containing premium offers were broadcast on non-children’s TV channels [OR = 0.45, 95 % CI (0.29, 0.71)]. Furthermore, among non-core food ads, promotional characters and premium offers were shown more on non-children’s TV channels compared with children’s TV channels [promotional characters: OR = 11.00, 95 % CI (5.53, 21.85); premium offers: OR = 6.66, 95 % CI (2.79, 15.85)]. Notably, the difference in premium offers in non-core food ads during non-peak time and peak time was marked, with an OR = 5.73 and 95 % CI (1.98, 16.59). Additionally, food ads containing promotion characters occurred less in the afternoon than in the morning [OR = 0.65, 95 % CI (0.50, 0.85)] while more non-core food ads contain premium offers in the afternoon and evening [afternoon: OR = 7.56, 95 % CI (2.52, 22.67); evening: OR = 3.88, 95 % CI (1.33, 11.28)].

**Table 4:** Characteristics of persuasive marketing techniques

	Food ads								Non-core food ads							
	Promotional characters				Premium offers				Promotional characters				Premium offers			
	*N*	*n* (%)	OR (95 % CI)	Adjusted OR (95 % CI)	*N*	*n* (%)	OR (95 % CI)	Adjusted OR (95 % CI)	*N*	*n* (%)	OR (95 % CI)	Adjusted OR (95 % CI)	*N*	*n* (%)	OR (95 % CI)	Adjusted OR (95 % CI)
Dates																
Weekdays	572	208 (36.4)	1.00 (−−)	1.00 (−−)	572	64 (11.2)	1.00 (−−)	1.00 (−−)	208	79 (38.0)	1.00 (−−)	1.00 (−−)	64	26 (40.6)	1.00 (−−)	1.00 (−−)
Weekends	837	334 (39.9)	1.16 (0.93,1.45)	0.93 (0.62,1.40)	837	90 (10.8)	0.96 (0.68,1.34)	0.82 (0.51,1.31)	334	201 (60.2)	2.47 (1.73,3.52)*	1.38 (0.52,3.66)	90	65 (72.2)	3.80 (1.93,7.50)*	3.31 (1.49, 7.37)*
Times																
Peak time	305	126 (41.3)	1.00 (−−)	1.00 (−−)	305	34 (11.2)	1.00 (−−)	1.00 (−−)	126	57 (45.2)	1.00 (−−)	1.00 (−−)	34	13 (38.2)	1.00 (−−)	1.00 (−−)
Non-peak time	1104	416 (37.7)	0.86 (0.66,1.11)	0.81 (0.60,1.10)	1104	120 (10.9)	0.97 (0.65,1.46)	1.08 (0.67,1.73)	416	223 (53.6)	1.40 (0.94,2.09)	1.14 (0.68, 1.92)	120	78 (65.0)	3.00 (1.37,6.59)*	5.73 (1.98, 16.59)*
Morning	457	197 (42.2)	1.00 (−−)	1.00 (−−)	457	54 (11.6)	1.00 (−−)	1.00 (−−)	197	106 (53.8)	1.00 (−−)	1.00 (−−)	54	26 (48.2)	1.00 (−−)	1.00 (−−)
Afternoon	528	172 (32.6)	0.66 (0.51, 0.86)*	0.65 (0.50, 0.85)*	528	49 (9.3)	0.78 (0.52, 1.18)	0.75 (0.49, 1.14)	172	94 (54.7)	1.04 (0.69, 1.56)	0.94 (0.59, 1.49)	49	38 (77.6)	3.72 (1.58, 8.77)*	7.56 (2.52, 22.67)*
Evening	414	173 (41.8)	0.98 (0.75,1.29)	0.95 (0.70,1.31)	414	51 (12.3)	1.08 (0.72,1.62)	1.05 (0.65,1.70)	173	80 (46.2)	0.74 (0.49,1.11)	1.11 (0.65, 1.88)	51	27 (52.9)	1.21 (0.56, 2.61)	3.88 (1.33, 11.28)*
Channels																
Central TV channels	1053	392 (37.2)	1.00 (−−)	1.00 (−−)	1053	132 (12.5)	1.00 (−−)	1.00 (−−)	392	209 (53.3)	1.00 (−−)	1.00 (−−)	132	82 (16.7)	1.00 (−−)	--
Local TV channels	356	150 (42.1)	1.23 (0.96,1.57)	0.75 (0.46,1.22)	356	22 (6.2)	0.46 (0.29,0.73)*	0.59 (0.29,1.20)	150	71 (47.3)	0.79 (0.54,1.15)	0.51 (0.18,1.47)	22	9 (40.9)	0.42 (0.17,1.06)	--
Children’s channels	478	124 (25.9)	1.00 (−−)	1.00 (−−)	478	85 (17.8)	1.00 (−−)	1.00 (−−)	124	20 (16.1)	1.00 (−−)	1.00 (−−)	85	35 (41.2)	1.00 (−−)	1.00 (−−)
Non-children’s channels	931	418 (44.9)	2.33 (1.83,2.96)*	2.64 (1.91,3.66)*	931	69 (7.4)	0.37 (0.26,0.52)*	0.45 (0.29,0.71)*	418	260 (62.2)	8.56 (5.10,14.4)*	11.00 (5.53,21.85)*	69	56 (81.2)	6.15 (2.93,12.92)*	6.66 (2.79, 15.85)*

*Ads* advertisements

*: *P* < 0.05

## Discussion

### Interpretation of total food ads

In our study, TV food advertising patterns were compared on five channels, and retail food and drink were the most frequently advertised products on TV (25.5 % of all ads), which was greater than the reported level globally (18 %) but less than that in Australia (31.1 %) and the entire Asia-Pacific area [20–22]. For all channels, the majority of food ads were for non-core foods (48.1 % of the food ads), and the percentage was less than in Hong Kong (58 %) and that reported globally (67 %) [21]. The most frequently advertised food products were milk, yoghurt and dairy products (24.7 % of the food ads). However, chocolate and confectioneries were the most advertised food products in Hong Kong (17 % of all food ads) and fast food restaurants occupied larger proportion of food products in Australia (14.5 % of all food ads) [21, 23]. Our study also found that there were no food ads simply promoting fruits and vegetables, which is similar to a previous study [24].

### Difference of food ads in viewing categories

In addition, the number of ads per hour per channel was 28.8, which is close to the level in the UK (28.2) [25]. Meanwhile, the frequency of food advertising was 6 food ads per hour per channel and this rate is lower than that in an international study of 13 countries (7 food ads per hour per channel) [21]. Based on our preliminary survey and previous study [26], we presume that if children watch TV an average of two hours per day, they will be exposed to 102 food ads containing 46 unhealthy food ads per week. It was demonstrated that TV viewing had a negative effect by stimulating the consumption of unhealthy foods while sitting still, and it also prevented the children from being physically active [27]. Other correlational researches have hypothesised the relation that TV food advertising can influence the food consumption and lifestyle patterns of children thus mediating the occurrence of childhood obesity [24, 28]. Therefore, reducing children’s contact with TV food advertising may have significance in weakening the risk of an obese environment.

Generally, CCTV-14 which was watched by the most children, contributed the highest proportion of food and non-core food ads among all channels. We speculated that in China, as central TV channels are considered to have greater levels of influence and trust among the public, marketers tend to choose these channels to achieve better publicity and long-term benefits so they broadcast more ads and food ads compared to local TV channels. Moreover, a larger proportion of food ads and non-core food ads were broadcast during non-children’s programmes than during children’s programmes. It is true that non-children’s programmes own a wider range of forms and categories, such as entertainment, movies and soup operas, so they can attract a greater audience, including both adults and children. Compared with the weekdays, almost all channels prefer to broadcast ads and food ads during the weekends, as found in a study in Singapore [29]. In China, It was demonstrated that weekends had higher audience density and more watching time [26] among adolescents. Moreover, children are likely to watch TV when they return home and prefer to eat snacks in the afternoon and evening [30], which is consistent with the more number of ads than in the morning.

### Difference of persuasive marketing techniques in food ads

Our results showed that the proportions of promotional characters and premium offers in food ads and non-core food ads were both higher than those reported in 13 other countries and in the Asia-Pacific area [20, 21]. Notably, these techniques were used the most in ads for non-core food products. Celebrities and giveaways are the dominant methods of promotion of advertised food on TV. Previous research showed that the promotional characters were not limited to fast food and confectioneries, but were also adopted by soft drink and other advertising [29]. In a recent study, children were more likely to prefer tasting and choosing for the first time food with popular cartoon characters compared with the same food without these characters [31].

Moreover, children often ask their parents to purchase the food that they have seen in TV ads, more importantly, the effects of TV viewing on food choices might have long-term effects persisting into young adulthood [32, 33]. It seems that a direct relationship exists between food advertising targeted to children and their food choices [34]. Strong evidence proves that food preferences and purchase requests from 2 to 11 year olds children are influenced by TV food advertising [35]. Therefore, restricting the content of food ads, especially for junk food, may be an effective method to reduce the impact of external risk factors for childhood obesity.

### Food advertising policies in China and other countries

While conducting this study, through relevant literature and data, we found that there were no specific regulations governing the mode of commercial food marketing in ads during children’s TV programming in China. This may be a defect in the management of food publicity regulation. In Germany and the UK, although the governments have formulated regulatory controls and do not allow advertisers to directly appeal to children in food ads, the use of promotional characters and other persuasive skills to appeal to children was still widespread in food advertising on popular channels [36], including indirect methods like cartoon characters [37]. Therefore, how to formulate and promulgate relevant laws and regulations and implement them effectively needs further studies.

### Limitations

Our findings also need to be viewed in the light of some limitations. To begin with, no significant difference in food ads was found during children’s peak viewing time and non-peak viewing time, while many more unhealthy food ads were screened during children’s non-peak viewing time than peak viewing time. We supposed that because the time partition was planned based on the high proportion of children’s TV-exposure time in the results of our preliminary study and it was different with the study of Zhou et al. and other countries [38, 11], the definition of children’s peak viewing time might be not accurate enough. Also, considering more unhealthy food ads were shown on non-children’s channels instead of the children’s, it can be concluded that perhaps unhealthy food ads are targeted not only at children but also at adults. Moreover, during the data collection process, the duration of ads was not considered but might be an influencing factor when children choose and consume food. Furthermore, the 5 selected channels were a portion of the TV channels selected by most children and they could not reflect all of the current advertising situation. Even so, these channels represented for different channel types including central/local TV and children’s/non-children’s TV, so they could partly reflect the difference among these channel types to some extent. Moreover, there were few researches of food advertising conducted in the northwest China at present. Considering the geographical distribution and economic level in China, our research in Xi’an can determine the characteristics of food advertising to children in the northwest of China to inform policies for the marketing improvement. More extensive research and investigation are needed, including a cohort study and a case-control study to obtain a better understanding of the conditions under which children of all ages are influenced by TV advertising and to reveal the relationship between childhood obesity and food advertising.

## Conclusions

Our research was a pilot study and the overall results indicated that there were high levels of food ads on TV, particularly for unhealthy foods compared to core foods and miscellaneous foods. In different viewing categories, more unhealthy food ads were shown in children’s non-peak time and afternoon as well as non-children’s channels. More children-oriented persuasive marketing tactics were used in unhealthy food ads especially in non-children’s channels. Therefore, findings from the current study guide more concerns and regulations. Public policy should govern the entrance of unhealthy food into the food market and establish regulations to restrict the advertising of unhealthy foods not only on children’s programmes/channels but also on non-children’s programmes/channels, and the promotion techniques in ads promoting unhealthy foods in order to prevent children’s exposure to unhealthy food marketing and childhood obesity.

## Abbreviations

ads, advertisements; CCTV-14, China Central Television-14; CCTV-5, China Central Television-5; CCTV-6, China Central Television-6; Hunan TV, Hunan Television; Shaanxi TV, Shaanxi Television; TV, television

## Additional file

Additional file 1: Table S1. Programme types for TV advertising spots. **Table S2.** Product types in TV advertising. **Table S3.** Food types in TV food advertising. (DOCX 23 kb)
