# Supplementary material for: The extent and nature of television food advertising to children in Xi’an, China
Source: BMC Public Health. 2016 Aug 11;16:770. doi: 10.1186/s12889-016-3468-0 (PMC4982426; doi:10.1186/s12889-016-3468-0)
Supplement: Additional file 1: Table S1. — Programme types for TV advertising spots. Table S2. Product types in TV advertising. Table S3. Food types in TV food advertising. (DOCX 23 kb) [file 12889_2016_3468_MOESM1_ESM.docx]

S1 Table Programme types of TV advertising spots

| Variable assignment | Programme types |  | Variable assignment | Programme types |
| --- | --- | --- | --- | --- |
| 1 | Comedies |  | 9 | Sports |
| 2 | Dramas |  | 10 | Entertainment |
| 3 | Movies |  | 11 | Documentaries |
| 4 | TV series |  | 12 | Games |
| 5 | Musicals |  | 13 | Children’s programmes |
| 6 | News/Newsletter reviews |  | 14 | Channel promotions |
| 7 | Talk shows |  | 15 | Others |
| 8 | Political events |  |  |  |

S2 Table Product types in TV advertising

| Variable assignment | Product types |
| --- | --- |
| 1 | Retail food and drink |
| 2 | Supermarkets/Markets |
| 3 | Restaurants (including nosheries and local bistros) |
| 4 | Clothes/Shoes |
| 5 | Education |
| 6 | Entertainment (such as movie, TV series, musical, variety show) |
| 7 | Economy (such as banks, insurance, social security) |
| 8 | Household cleaning products (such as laundry detergents, detergents) |
| 9 | Home appliances |
| 10 | Transportations (such as electric bicycles, cars, fuel) |
| 11 | Pets (including pet foods) |
| 12 | Chemical drugs (such as medicines、vitamins、air fresheners) |
| 13 | Public service (general) |
| 14 | Public service (business) |
| 15 | Literatures (such as magazines, newspapers, books) |
| 16 | Retailing |
| 17 | Toiletries |
| 18 | Toys |
| 19 | Tourism |
| 20 | Utilities equipment (such as water, electricity, telephones) |
| 21 | Channel promotions (including channels and other programme promotions) |
| 22 | Electronic products (such as computers, mobile phones) |
| 23 | Others |

S3 Table Food types in TV food advertising

| Food types | Variable assignment | Food categories |
| --- | --- | --- |
| Core/healthy food | 1 | Bread, rice and rice products (without fat, sugar and salt), noodles (non-fried), pure starch products, ordinary biscuits and biscuits |
|  | 2 | Low-sugar, high-fibre breakfast cereal (< 20 g sugar/100 g food, > 5 dietary fibre/100 g food) |
|  | 3 | Fruits and fruit products (no additives such as fresh and canned original  juices, dried fat, sugar or salt), juice content ≥ 98% fruit |
|  | 4 | Vegetables and vegetable products ((no additives such as fresh, canned, dried fat, sugar or salt), including pure seaweeds |
|  | 5 | Milk, yogurt, cheese and other dairy products, including probiotic drinks |
|  | 6 | Meat and meat substitutes, including meat, poultry, fish, beans, tofu, eggs and raw, fresh nuts |
|  | 7 | Enriched in monounsaturated fats or polyunsaturated fats oils, such as  olive oil, sunflower oil, soya oil, plant-based margarine and its  extension products, and low-fat, tasty sauces (< 10 g fat/100 g) |
|  | 8 | Low-fat and low-salt diet, including frozen or packaged food (≤ 6 g saturated fat/bag, ≤ 900 mg sodium/bag)，soup (< 2 g fat/100 g, except dehydration), sandwiches, mixed salads, steamed bread (except sweet bread), dumplings without frying |
|  | 9 | Healthy snacks, based on core and healthy foods (such as fruit, vegetables, cereals, dairy, beans, meat and its substitutes), energy content < 600 KJ/bag, < 3 g saturated fat/bag, < 200 mg sodium/bag |
|  | 10 | Baby products (except milk formula) |
|  | 11 | Bottled water (including mineral water of no taste and soda water) |
| Non-core/  unhealthy food | 12 | High-sugar and/or low-fibre breakfast cereals (> 20 g sugar/100 g or < 5 g dietary fibre/100 g) |
|  | 13 | Flavored or fried instant rice and pasta |
|  | 14 | Sweet bread, cakes, muffins, donuts (such as lotus seed, cream and red bean filling), sweet biscuits (including omelettes), sweet glutinous rice balls or cakes, high-fat pies, biscuits and pastries, sweet glutinous rice and rice pudding |
|  | 15 | Meat and meat substitutes with salt treatment or saving, including crab  sticks, jellyfish salads, canned meat and all the meat, poultry, fish, tofu  and egg products that were preserved and ready to eat |
|  | 16 | Sweet snacks, including jelly, sugar-coated nuts, nut- and seed-based milks, sweet rice wine, canned fruit |
|  | 17 | Tasty snacks (added salt or fat), including chips, spicy dry peas, dried  fruit slices, extruded snacks, popcorn (except unprocessed popcorn),  salty nuts, and other fried snacks |
|  | 18 | Fruit juices/Drinks (< 98% fruit) |
|  | 19 | Ice cream, ice candies, and desserts |
|  | 20 | Chocolates and candies, including cotton candies, candies (all types) and chewing gums (except sugar-free varieties) |
|  | 21 | Fast food, such as hamburgers, French fries and beverages |
|  | 22 | High-fat or high-salt diet, frozen or package food (> 6 g saturated fat/bag, > 900 mg sodium/bag), steamed bread, steamed buns (except sweet bread), pan-fried dumplings and Chinese ravioli |
|  | 23 | Other high-fat or high-salt food-including meat, fish, bean paste, salad  dressing, butter and animal fat, high-fat tasty sauces (> 10 g fat/100 g),  soup (> 2 g fat/100 g, dehydrated) |
|  | 24 | Sugar sweetened drinks, including soft drinks, sweet tea drinks, sports/electrolyte drinks, beverage powder addition (such as Nestle coffee, sweet tea or sweet coffee powder) |
|  | 25 | Wine |
| Miscellaneous  food | 26 | Other food additives (including chicken powder, oil, dry spices, seasoning) |
|  | 27 | Vitamins, minerals or dietary supplements, sugar-free chewing gums |
|  | 28 | Tea and coffee (except sweet tea or sweet coffee) |
|  | 29 | Baby and toddler milk formula |
|  | 30 | Fast food (only healthy choices), such as chicken roulade, water and  fruit slices |
|  | 31 | Fast food shops (without food or drinks) |
|  | 32 | Local restaurants |
|  | 33 | Supermarkets (only core and healthy foods) |
|  | 34 | Supermarkets (with core, healthy foods and other foods) |
|  | 35 | Supermarkets (without food or drinks) |
